# Isolation, Characterization and Selection of Avermectin-Producing *Streptomyces* *avermitilis* Strains From Soil Samples

**DOI:** 10.5812/jjm.10366

**Published:** 2014-06-01

**Authors:** Samia Siddique, Quratulain Syed, Ahmad Adnan, Fahim Ashraf Qureshi

**Affiliations:** 1 Department of Chemistry, Government College University, Lahore, Pakistan; 2 Food and Biotechnology Research Center, Pakistan Council of Scientific and Industrial Research Laboratories Complex Ferozepur, Lahore, Pakistan; 3 Office of Research, Innovation and Commercialization, Comsats Institute of Information and Technology, Islamabad, Pakistan

**Keywords:** *Streptomyces*, Anti-Infective Agents, Avermectin

## Abstract

**Background:**

*Streptomyces avermitilis,* belonging to *Actinomycetes, *is specialized for production of avermectin, used as an anthelmintic and insecticidal agent. It is mostly found in soil and its isolation is very crucial for medically important avermectin production.

**Objectives:**

In the present study, 10 bacterial isolates lacking antimicrobial activities were isolated from the soil samples collected from different areas of Lahore, Pakistan.

**Materials and Methods:**

Three distinctive localities of Lahore were opted for soil assortment to isolate *S. avermitilis*. About 50 isolates of *Streptomyces* species were attained through selective prescreening procedures. All of these isolates were studied for production of the secondary metabolite, avermectin. Different test like soluble pigment color and melanin formation were used for identification. Biochemical characterizations of those isolates closely resembling the control in morphological characteristics, soluble pigment color and melanin formation tests were performed.

**Results:**

The 10 selected isolates were identified as the avermectin-producing strain by fermentation and characterized on ISP2 medium for aerial and reverse side mycelia color, soluble pigment color and melanin formation, in comparison with *S. avermitilis* DSM 41445. The best avermectin-producing isolate S1-C (10.15 mg/L) showed similar result as *S. avermitilis* DSM 41445, when subjected for culture characteristics analysis in different media along with biochemical characterization.

**Conclusions:**

From the results, it was concluded that agricultural lands around Pakistan Council of Scientific and Industrial Research (PCSIR) Campus Lahore were rich sources of industrially important *Streptomyces,* especially *S. avermitilis*.

## 1. Background

*Streptomyces,* belonging to the most profuse group of microorganisms in soil, the *Actinomycetes,* are aerobic and Gram-positive bacteria (1, 2). Their distribution and presence in soil is highly affected by geographical conditions of the soil like temperature, type, pH, amount of organic materials, and moisture contents. The acidic environment-resistant groups are the most abundant of all *Actinomycetes* in the soil (3). However, they are less abundant in soils with alkaline pH (4) and are famous for their ability of producing industrially important enzymes and secondary metabolites during the fermentation process (5) as well as covering about 80% of antibiotic products (6). Screening and isolation of microorganisms producing secondary metabolites have been the main focus for several years (7).

Components of media affect the *Streptomyces *isolation. Media containing glycerol or starch as carbon sources and arginine, casein or nitrate as nitrogen sources will result in the best isolation. Different antifungal agents named nystatin, cycloheximide and pimaric in are usually employed during the isolation to obtain pure bacterial isolates. Identification of *Streptomyces* is based on the spore size, morphology, chains, pigmentation, physiological and biochemical characteristics, and antibiotic resistance (8).

Standard microbiological methods, analysis of biochemical markers, and DNA sequencing have also been employed for selective identification of genus and species of the isolated microbes (9). *Streptomyces* form stable filaments and are also capable of producing long chains of spores with aerial growth. Direct and non-direct methods of screening antibiotic producing strains have usually been employed for isolating a specific microbe. Direct screening of strains involves bio assay or some chemical methods, while non-direct screening involves correlation of the strain characteristics with antibiotic production (10-12). *Streptomyces *members are very important because of their ability to produce several types of secondary metabolites (13). 

*Streptomyces avermitilis*, a Gram-positive bacteria, is specialized for formation of secondary metabolites, from which avermectinis used as an anthelmintic agents. Avermectin is one of the 16-membered macrocyclic lactone derivatives with anthelmintic and insecticidal potential, generated from *S. avermitilis* isolated from soil. It is used to control insects and mite pests of a range of agronomic, fruit, vegetable and ornamental crops. The main use of avermectin is controlling fire ants. Ivermectin, as emisynthetic derivative of avermectin, is widely used in veterinary for improved animal health as well as on chocerciasis eradication (14-16).

## 2. Objectives

The present study was conducted for isolation of *S. avermitilis* from the soil samples of different locations of Pakistan Council of Scientific and Industrial Research (PCSIR), Lahore, Pakistan, using various pretreatment methods. Different media were employed for screening of pure *Streptomyces* species. Antibacterial and antifungal activities of various isolates were also evaluated.

## 3. Materials and Methods

### 3.1. Collection and Preparation of Soil Samples

Different locations of PCSIR Lahore and Punjab University were selected for soil sampling. About 3 cm of each soil surface was removed with sterilized spatula and samples were stored in clean, dry and sterilized polythene bags at 40°C until pretreatment (17). For each soil sample, 1g of soil was suspended in 100 mL of sterile saline and incubated at 28°C in orbital shaker for 30 minutes at 150 rpm. Soil samples were vortexed with the maximum speed and then allowed to stand for a few minutes. Serial dilutions up to 10-5 of each soil sample were made in sterile saline (18). For separation of spores from vegetative cells, test tube of dilution 10-5 was placed in a 45°C-water bath for 16 hours (13).

### 3.2. Isolation of Streptomyces Colonies From the Soil Samples

The media employed for isolation and enumeration of *Actinomycetes *were *Actinomycete* isolation agar medium (19): 5 g/L glycerol, 4 g/L sodium propionate, 2 g/L sodium casemate, 2 g/L KH_2_PO_4_, 0.1 g/L asparagine, 0.1 g/L MgSO_4_.7 H_2_0,1 mg FeSO_4_.7 H_2_O, 15 g/L agar, pH = 7.0; Kuster’s Agar medium (20): 10 g/L Glycerol, 0.3 g/L casein, 3 g/L KNO_3_, 2 g/L K_2_HPO_4_, 2 g/L NaCl, 0.05 g/L MgSO_4_.7H_2_O, 0.02 g/ LCaCO_3_, 0.01 g/L FeSO_4_.7H_2_O, 16 g/L agar, pH = 7 ± 1; glycerol casein KNO_3 _agar medium (1): 10 g/L Glycerol, 0.3 g/L casein, 2 g/L KNO_3_, 2 g/LK_2_HPO_4_, 2 g/L NaCl, 0.05 g/L MgSO_4_.7H_2_O, 0.02 g/LCaCO_3_, 0.01 g/L FeSO_4_.7H_2_O, 18 g/L agar, pH = 7.8; and starch casein agar medium (21): 10 g/L starch, 1 g/L casein powder, 15 g/L agar, 50% sea water, pH = 7.2± 0.2. Each of these media was supplemented with nystatin at concentration of 0.050 mg/mL as the antifungal agent (22, 23).

Dilutions of each soil sample were spread on the plates containing the isolation media and incubated at 28°C for 7-10 days. Individual colonies from the mixed cultures were then transformed onto yeast extract-malt extract agar slants (13). Pure cultures were obtained by multiple streaking on yeast extract malt extract glucose medium (YMG) agar slants (24): yeast extract, 4 g/L; malt extract, 10 g/L; glucose, 4 g/L; agar, 20 g/L; pH = 7.3. Finally, the *Streptomyces* isolation medium consisting of glucose: 5.0 g/L, L-glutamic: 4.0, KH_2_PO_4_: 1.0, MgSO_4_.7H_2_O: 0.7, NaCl: 1.0, FeSO_4_.7H_2_O: 3.0 mg, and agar: 25 g/L, was used for purification of *Streptomyces* colonies (20) supplemented with nystatin at concentration of 0.050 mg/mL as the antifungal agent (22, 23).

### 3.3. Study of Antimicrobial Activities

Antimicrobial activities of the isolated *Streptomyces* strains were studied against different bacteria (*Escherichia coli*, *Enterobacter aerogenes*, *Staphylococcus. aureus*, *Pseudomonas aerogenosa *and *Bacillus subtilis*), fungi (*Aspergillus niger*, *Rhizopus oligosporus*) and yeast (*Candida albicans*). All these strains were obtained from the Food and Biotechnology Department of PCSIR and cultured in nutrient broth for 24 hours at 37^°^C (13). For yeast and fungi cultures, the incubation period was five days.

Well diffusion method was used for studying antimicrobial activities of the isolates. Wells were separately created in each plate using the sterilized borer, already seeded with 300 µL of the test organisms (25). Each well was then filled with 300 µL of supernatant of each isolate dilution and kept for at least one hour to ensure the complete diffusion of dilution into the nutrient agar medium in each plate. Diameters of the zones, formed after the incubation periods of 24 and 48 hours at 37^°^C, were measured (19). Control plates were also made, empty of the isolated strains, to investigate the normal growth of *Streptomyces*. Plates of the reference *S. avermitilis* DSM 41445 were also prepared for comparative analysis.

### 3.4. Characterization of the Isolated Colonies

#### 3.4.1. Gram Staining

Gram staining of the isolates was performed according to the method described earlier (18).

#### 3.4.2. Avermectin Production

All the purified isolates were tested for production of avermectin through fermentation, along with *S. avermitilis* DSM 41445 for comparative study.

### 3.5. Seed Medium

The cultures were maintained on medium 65 (glucose: 4.0 g/L, yeast extract: 4.0 g/L, malt extract: 10.0 g/L, CaCO_3_: 2.0 g/L, and agar: 12 g/L) as specified by Deutsche Sammlung von Mikroorganis men und Zellkulturen (DSMZ) Loopful culture of the strain was scraped from the nutrient agar slant and inoculated into 50mL of yeast malt glucose (YMG) medium consisting of glucose: 4.0, yeast extract: 4.0, malt extract: 10.0 and CaCO3: 2.0 (g/L in distilled water) in a 250-mL shake flask. After wards, it was incubated in an orbital shaker at 150x g for 16-18hours at 30^°^C in a water bath shaker (Eyela, Japan). pH of the inoculum was adjusted at 7.2 ± 2 (26).

### 3.6. Avermectin Production

Production of avermectin B1b from the soil isolates was studied individually in synthetic medium 2 (SM2) growth medium. Each production medium was inoculated with 5 mL (10%v/v) of inoculum medium separately. After transferring the seed medium, each growth medium was incubated at 30^°^C in the water bath shaker for 10 days at 150x g. Composition of the growth medium was soluble corn starch: 50.0, KCl: 0.1, NaCl: 0.5, yeast extract: 2.0, MgSO_4_.7H_2_O: 0.1, CaCO_3_: 0.8 and α-amylase: 0.1 (all in g/L). pH of the medium was adjusted at 7.2 ± 0.2. All the experiments were separately performed in the shake flasks containing 50mL of the fermentation medium (26).

### 3.7. Extraction of Avermectin B1b

The fermentation broth from each fermentation flask was centrifuged (H-1500FR Japan) at 40°C for 20 minutes at 8000x g. Since avermectin is an intracellular molecule, the cell biomass was separated and the supernatant was discarded. The cell biomass in the form of pallet was mixed with an appropriate amount of methanol to completely dissolve it. The mixture was centrifuged again and the supernatant was collected for avermectin analysis by high-performance liquid chromatography HPLC (26).

### 3.8. HPLC Analysis of Avermectin B1b

Concentrations of the avermectin components were determined quantitatively by reverse phase HPLC (LC-2080, Shimadzu, India). About 20 µL of each sample was run into the HPLC. The samples were separated on C18 column (SMA C-18) and detector (UV Variable Wavelength Detector STD-M20A, Shimadzu, India) and eluted by methanol: acetonitrile (98: 2 v/v) at a flow rate of 0.5 mL/min with a UV absorbance at 246 nm (27).

#### 3.8.1. Biological Testing

The isolates with avermectin B1b production were further identified by certain biological tests including growth temperature range test, hemolysis test, urea hydrolysis, oxalate utilization test, H_2_S production test, acid production test, carbohydrate assimilation test, amylolytic activity and proteolytic activity, as recommended by International *Streptomyces* Project (ISP). Utilization of different carbon sources such as glucose, starch, mannitol, fructose, soluble corn starch, potato starch and maltose, and nitrogen sources namely malt extract, yeast extract, peptone, urea and lemco powder were also tested on *Streptomyces* isolation medium (19). The medium was supplemented with nystatin at concentration of 0.050 mg/mL as the antifungal agent (22, 23). Plates of the reference culture of DSM *S. avermitilis* 41445 were also prepared for comparative study.

## 4. Results

Only 10 soil isolated strains were found to lack antimicrobial properties. These 10 isolates, when tested for secondary metabolite production through submerged fermentation, were capable of avermectin production as is shown in Table 2. The soil isolate named S1-C gave maximum (10.15 mg/L) avermectin production. Morphological characteristics of these isolates revealed that they closely resembled *S. avermitilis* species, when compared with the control strain *S. avermitilis* DSM 41445. Therefore the 10 isolates were grouped as *S. avermitilis*. The percentage of color production by these 10 isolates in the form of soluble pigments, varied within a color series. Production of soluble pigments in dark yellow, yellow, pale yellow and brownish yellow was about 10%, 60%, 20% and 10%, respectively. Rate of melanin production for these 10 isolates was 100%. The aerial mycelium color was dark grey (10%), grey (70%) and light grey (20%). Similarly, the reverse side color was moderate grey (10%), grey (50%), light grey (20%), and white to grey (20%). 

Table 3 reveals that in oatmeal agar medium (ISP3) and yeast extract-malt extract agar (ISP2), S1-C and control showed same characteristic. In nutrient agar medium, both strains showed same growth patterns. The difference was in the color of aerial mycelium and the reverse side color, which was also very minute. In other media, the color ranged from grey to light grey. In DSMZ (Deutsche Sammlung von Mikroorganismen and Zellkulturen) specified medium 65, color varied from yellow to light yellow with excellent growth of control as compared to the isolated strain showing growth of good level.

## 5. Discussion

The present study was conducted for isolation of *Streptomyces* from the soil samples taken from different locations of PCSIR and other locations of Lahore. The main emphasize was on selection of *Streptomyces* spp., producing the avermectins as anthelmintic agent.

About 50 soil samples were collected for isolation of the desired *Streptomyces*. All the isolated were tested against eight test microorganisms, three of which were Gram-negative (*E. aerogenes*, *E. coli*, *P. aerogenosa*) and two Gram-positive (*S. aureus*, *B. subtilis*). Two fungal species (*R. oligosporus*, *A. niger*) and one yeast (*C. albicans*) were also used to test the antimicrobial activities of the isolates. Patterns of antimicrobial activities of all these isolated are given in Table 1 showing that 10 isolates were completely resistant to all the test microbes and did not show any activity. It was reported in an earlier research that *S. avermitilis* lacks characteristic antimicrobial activities (28). In the present study, only 10 did not show antimicrobial activities. These strains were supposed to be *S. avermitilis,* producing the avermectin compound, lacking antibacterial and antifungal activities. The control plates were also prepared for comparison and confirmatory studies.

All the 10 isolates belonged to the *Streptomyces* genus, when examined morphologically on ISP 2 Yeast extract-malt extract medium (13, 24). In a previous study, researchers found that *Streptomyces* isolates showed 13.3% melanin pigment production. Furthermore, the color variation for aerial mycelium was brown-yellow (80%), yellow (6.6%) and violet (13.3%). They noticed 93.3% brownish-yellow and 6.6% violet in reverse side mycelium (13). The morphological characterization of *Streptomyces* isolates normally relied upon the formation and color of aerial and substrate mycelium, soluble pigment formation, and spore characteristics (8).

The fermentation broth obtained during secondary metabolite production lacked antibacterial and antifungal activities. It is reported that avermectins are a series of macrocyclic lactones, lacking significant antibacterial and antifungal activities (29, 30). In another research, it was reported that *S. avermitilis *was specialized for its ability to produce secondary metabolites, which was potentially anthelmintic and named avermectin (1, 30). In the present study, all the 10 strains were then tested for production of secondary metabolite through fermentation. It was found that these isolates produced avermectin in very small quantities, as is shown in Table 2. The maximum avermectin production (10.15 mg/L) was presented by the soil isolate designated as S1-C.

Culture characteristics of *S. avermitilis* 173 on different media were observed, which resulted in different growth patterns and mycelia colors (30). In the present study, the same variations were observed. Isolated S1-C strain showed the maximum avermectin production and was selected for studying the cultural characteristics on different media along with the control strain DSM *S. avermitilis*. 

Morphological and biochemical characteristics derived from the physiological test, have been used for taxonomic classification and identification of different strain of *Streptomyces *(20, 31). In the present study, selective avermectin-producing S1-C *Streptomyces* species were subjected to different biochemical tests for identification, and later they were compared with that of the control strain, as shown in Table 4. The S1-C strain isolated at mesophilic temperature (25-37°C) was in agreement with results of other researchers, showing the isolation of most of the *Streptomyces* to be appropriate at these conditions. Production of avermectin from S1-C isolate at neutrophilic medium with pH range of 7.0-7.5 confirmed the strain dependency to *Streptomyces*(8).The S1-C strain and control showed same results in all biochemical tests.

**Table 1.** Antimicrobial Activities of *Streptomyces* Isolates ^a^

Zone of Inhibition, mm									
*Streptomyces *Isolation Agar Medium									
Serial Number	Soil Isolate	*Escherichia coli*	*Enterobacter aerogenes*	Staphylococcus *.aureus*	*Pseudomonas . aerogenosa*	Bacillus *.subtilis*	*Aspergillus. niger*	Rhizopus *. oligosporus*	*Candida . albicans*
**1**	S1-A	-	-	-	-	+	-	-	-
**2**	S1-B	+	+	+	-	-	-	+	+
**3**	S1-C	-	-	-	-	-	-	-	-
**4**	S1-D	-	-	+	+	++	-	-	-
**5**	S1-E	-	-	++	-	+	++	+	+
**6**	S1-F	++	+	-	+++	-	-	+	+
**7**	S1-G	-	-	+	-	+	-	-	-
**8**	S1-H	-	-	-	+	-	++	-	-
**9**	S1-I	++	++	+	+	-	-	+	+
**10**	S1-J	-	-	-	-	-	-	-	-
**11**	S2-A	-	-	-	+	+	-	++	-
**12**	S2-B	-	-	++	-	-	-	-	+++
**13**	S2-C	+	-	-	+	-	++	-	-
**14**	S2-D	-	+	-	-	+			
**15**	S2-E	-	-	-	-	-	-	-	-
**16**	S2-F	-	-	+	-	-	+	-	-
**17**	S2-G	-	+	++	+	-	-	-	+
**18**	S2-H	+	-	+	-	-	-	++	-
**19**	S2-I	+	-	+	-	+	-	-	++
**20**	S2-J	-	-	-	-	-	-	-	-
**21**	S3-A	-	-	+	-	+	-	+	-
**22**	S3-B	-	-	+	-	++	-	+	-
**23**	S3-C	++	+	-	+	-	++	-	+
**24**	S3-D	++	+	-	+	-	++	-	-
**25**	S3-E	-	-	-	-	-	-	-	-
**26**	S3-F	-	-	+	-	+	-	+	-
**27**	S3-G	-	+	+	+	-	-	-	-
**28**	S3-H	-	++	-	-	-	+	+	-
**29**	S3-I	-	-	-	-	-	-	-	-
**30**	S3-J	++	+	-	-	+++	-	-	+
**31**	S4-A	+	++	++	-	+	-	-	+
**32**	S4-B	-	-	+	++	-	+	+	-
**33**	S4-C	+	+	++	-	-	-	-	+
**34**	S4-D	++	+	-	+	-	++	-	+
**35**	S4-E	+	-	-	+	-	++	-	-
**36**	S4-F	-	-	-	-	-	-	-	-
**37**	S4-G	+	-	-	+	-	++	-	-
**38**	S4-H	+	-	++	-	+	-	-	+
**39**	S4-I	+	++	++	-	+	-	-	+
**40**	S4-J	-	-	-	-	-	-	-	-
**41**	S5-A	+	-	-	+	-	++	-	-
**42**	S5-B	++	+	-	+	-	++	-	+
**43**	S5-C	-	-	-	-	-	-	-	-
**44**	S5-D	+	++	++	-	+	-	-	+
**45**	S5-E	+	-	+	++	+	-	-	+
**46**	S5-F	++	+	-	+++	-	-	+	+
**47**	S5-G	+	-	-	+	-	++	-	-
**48**	S5-H	-	-	-	-	-	-	-	-
**49**	S5-I	+	-	++	-	+	-	-	+
**50**	S5-J	++	+	-	+	-	++	-	+
**51**	Control	-	-	-	-	-	-	-	-

^a^ + = Fair,++ = potent,+++ = highly potent,- = no effect.

**Table 2.** Secondary Metabolite (Avermectin) Production of Selected Isolates ^a,b^

Serial Number	Soil Isolate	Avermectin Production, mg/L
**1**	S1-C ^c^	10.15±0.04
**2**	S1-J	5.0±0.05
**3**	S2-E	7.35±0.01
**4**	S2-J	6.29±0.09
**5**	S3-E	8.35±0.06
**6**	S3-I	6.0±0.011
**7**	S4-F	3.86±0.02
**8**	S4-J	5.78±0.01
**9**	S5-C	5.64±0.02
**10**	S5-H	8.65±0.07
**11**	Control	17.0±0.03

^a^ Data are presented as mean ± SD.

^b^ Shake flask fermentation at pH 7.0, temperature: 31°C. Each value is an average of three replicates.

^c^Designation of the best avermectin producing strain.

**Table 3.** Cultural Characteristics of S1-C Strain on Different Media

Serial Number	Type of Medium	Growth		Aerial Mycelium		Reverse Side Color	
		S1-C	Control	S1-C	Control	S1-C	Control
**1**	Nutrient agar	Good	Good	Dark yellow	Light yellow	Light yellow	White to yellow
**2**	Yeast extract malt extract agar (ISP2)	Good	Good	Dark grey	Dark grey	Moderategrey	Moderate to light grey
**3**	Inorganic salt-starch agar (ISP4)	Moderate	Excellent	Grey	Dark grey	Light cinnamon	Light grey
**4**	PDA agar	Moderate	Good	White to yellow	Dark yellow	Light yellow	Pale yellow
**5**	Oatmeal agar (ISP3)	Good	Good	Grey	Grey	White to grey	White to grey
**6**	Bennett’s agar	Good	Very Good	Grey	Grey	Light grey	Light grey
**7**	Casein enzymichydrolysate-yeast extract (ISP1)	Good	Good	Light grey	Light grey	Light grey	White to grey
**8**	DSMZ medium 65	Good	Excellent	Yellow	Light yellow	Light yellow	White to yellow

**Table 4.** Morphological and Biochemical Characteristics of the Selected Avermectin-Producing S1-C *Streptomyces*

Serial Number	Properties	S1-C *Streptomyces* spp.	Control
**A**	Morphological characteristics		
1	Spore morphology	Oval shaped, smooth	Smooth
2	Color of aerial mycelium	Dark grey	Grey
3	Color of substrate mycelium	Grey	Light grey
4	Gram’s reaction	Gram positive	Gram positive
**B **	Biochemical characteristics		
1	Growth temperature range	28-37°C	28-37°C
2	pH range	7.0-7.5	7.0-7.5
2	Nitrate reduction test	-	-
3	Milk coagulation and peptonization	+	+
4	Gelatin liquefication	+	+
5	H_2_S production test	-	-
6		
	N-source utilization		
	Yeast extract	+	++
	Malt extract	+	+
	Peptone	+	+
	Urea	+	+
	Lemco powder	+	+
7		
	C-source utilization		
	Glucose	+	+
	Soluble corn starch	++	++
	Maltose	+	+
	Lactose	+	+
	Mannitol	+	+
	Wheat powder	+	+
	Potato starch	++	++
